# Pre-Analytical Conditions in Non-Invasive Prenatal Testing of Cell-Free Fetal *RHD*

**DOI:** 10.1371/journal.pone.0076990

**Published:** 2013-10-18

**Authors:** Frederik Banch Clausen, Tanja Roien Jakobsen, Klaus Rieneck, Grethe Risum Krog, Leif Kofoed Nielsen, Ann Tabor, Morten Hanefeld Dziegiel

**Affiliations:** 1 Department of Clinical Immunology, Section 2034, Copenhagen University Hospital, Rigshospitalet, Copenhagen, Denmark; 2 Center of Fetal Medicine, Department of Obstetrics, Copenhagen University Hospital, Rigshospitalet, Copenhagen, Denmark; 3 Faculty of Health Sciences, University of Copenhagen, Copenhagen, Denmark; German Red Cross Blood Service Frankfurt, Germany

## Abstract

**Background:**

Non-invasive prenatal testing of cell-free fetal DNA (cffDNA) in maternal plasma can predict the fetal RhD type in D negative pregnant women. In Denmark, routine antenatal screening for the fetal RhD gene (*RHD*) directs the administration of antenatal anti-D prophylaxis only to women who carry an RhD positive fetus. Prophylaxis reduces the risk of immunization that may lead to hemolytic disease of the fetus and the newborn. The reliability of predicting the fetal RhD type depends on pre-analytical factors and assay sensitivity. We evaluated the testing setup in the Capital Region of Denmark, based on data from routine antenatal *RHD* screening.

**Methods:**

Blood samples were drawn at gestational age 25 weeks. DNA extracted from 1 mL of plasma was analyzed for fetal *RHD* using a duplex method for exon 7/10. We investigated the effect of blood sample transportation time (*n* = 110) and ambient outdoor temperatures (*n* = 1539) on the levels of cffDNA and total DNA. We compared two different quantification methods, the delta Ct method and a universal standard curve. PCR pipetting was compared on two systems (*n* = 104).

**Results:**

The cffDNA level was unaffected by blood sample transportation for up to 9 days and by ambient outdoor temperatures ranging from -10°C to 28°C during transport. The universal standard curve was applicable for cffDNA quantification. Identical levels of cffDNA were observed using the two automated PCR pipetting systems. We detected a mean of 100 fetal DNA copies/mL at a median gestational age of 25 weeks (range 10–39, *n* = 1317).

**Conclusion:**

The setup for real-time PCR-based, non-invasive prenatal testing of cffDNA in the Capital Region of Denmark is very robust. Our findings regarding the transportation of blood samples demonstrate the high stability of cffDNA. The applicability of a universal standard curve facilitates easy cffDNA quantification.

## Introduction

Non-invasive prenatal testing (NIPT) has translated into several clinical applications based on the analysis of cell-free fetal DNA (cffDNA) obtained from the plasma of pregnant women [1]–[8]. Predicting the fetal RhD (D) type in D negative women helps to identify pregnancies at risk of D alloimmunization. D immunization may lead to hemolytic disease of the fetus and the newborn (HDFN), a condition that can result in fetal anemia, hydrops fetalis, jaundice, kernicterus, and intrauterine death [9]. HDFN prevention and early management is an important obstetric goal to minimize neonatal mortality and morbidity.

In 1997, cffDNA was discovered in the maternal circulation [10]. In 1998, it was demonstrated that the fetal RhD gene (*RHD*) could be detected in blood from pregnant D negative women who gave birth to D positive infants [11], [12]. In 2001, Finning and colleagues introduced the first real-time PCR-based clinical service for detecting fetal *RHD* to assist in the management of pregnancies of D immunized women [13], [14]. Since then, large-scale studies have demonstrated the feasibility of real-time PCR-based, high-throughput, routine screening for fetal *RHD* to guide targeted, routine antenatal anti-D prophylaxis [15]–[17]. Consequently, antenatal anti-D can be restricted to those D negative women who carry a D positive fetus, thus avoiding unnecessary antenatal treatment and use of anti-D immunoglobulin. In 2010, the first nationwide antenatal screening for fetal *RHD* was implemented for clinical use in Denmark, and a sensitivity of 99.9% was reported for the first six months of routine screening [18]. A nationwide screening program was launched in the Netherlands in 2011, and a preliminary evaluation showed that false negative results were reported in <0.25% [19]. A Swedish study of routine screening in early pregnancy showed a sensitivity of 98.9% when analyzing samples from as early as gestational age (GA) 8 weeks and onward [20].

A major concern for NIPT is the risk of false negative results resulting from the very small quantities of cffDNA present in the maternal plasma [21]. False negative results predominantly occur early in pregnancy [20], [22]–[27], but they have also been described later in gestation [23], [28], [29]. The consequence of a false negative result may be critical, as the pregnant woman will not receive Rhesus prophylaxis and may give birth to an infant affected by HDFN. This emphasizes that assay sensitivity and robustness is critical to the reliable detection of cffDNA. Several pre-analytical factors may influence the analytical outcome, including the transportation of blood samples [30], the handling and storage of samples [31], and the efficiency of the DNA extraction [32]–[35].

In this study, selected aspects of the antenatal *RHD* screening setup in the Capital Region of Denmark were evaluated in detail. We investigated whether blood sample transportation time and/or ambient outdoor temperatures during transportation would affect the detection of cffDNA. Our study used clinical samples from a routine analysis, as opposed to samples investigated under controlled laboratory conditions. We tested different real-time PCR-based methods for detecting and quantifying cffDNA, and we evaluated clinical aspects of the prophylaxis program.

## Materials and Methods

### Ethics statement

This study was undertaken as part of a quality assurance program for the antenatal *RHD* screening analysis of the Capital Region of Denmark. Routine blood sampling for routine antenatal *RHD* screening was taken with informed consent. Additional testing for *RHD* using residual blood material was approved by the Scientific-Ethical Committees for Copenhagen and Frederiksberg (KF 01283691) which waived the need for written consent. The database studies were approved by the Danish Data Protection Agency, according to the Danish Law on Research Ethics in health research.

### Blood samples

Blood samples from pregnant D negative women in the Capitol Region of Denmark were collected in 6-mL EDTA tubes at a routine visit to the general practitioner at GA 25 weeks. As part of the Danish national antenatal *RHD* screening program [18], the blood samples were analyzed at the Laboratory of Blood Type Genetics, the Department of Clinical Immunology, Rigshospitalet, the centralized laboratory for the antenatal analysis of blood samples from the Capital Region of Denmark. The samples were collected and analyzed in 2010. Samples only from non-immunized RhD negative women were tested. All samples were subjected to a visual inspection for hemolysis, and hemolyzed samples were discarded and new samples were requested.

### GA at blood sampling

GA at blood sampling was calculated using the date of birth, which was retrieved from the Danish Fetal Medicine Database; the GA at birth based on CRL measurement in the 1^st^ trimester ultrasound scan; and the date of blood sampling.

### DNA extraction

Blood samples were centrifuged at 1700× *g* for 10 min. Automated DNA extraction was conducted from 1 mL plasma using the QIAsymphony SP instrument (Qiagen Inc., Basel, Switzerland) and the QIAsymphony Virus/Bacteria Midi Kit with carrier RNA. Centrifuged blood samples were placed directly in the QIAsymphony SP without a second plasma centrifugation. The elution volume was 60 µL. From previous experience, automated DNA extraction was as efficient as manual extraction with the QIAamp DSP Virus Kit used as previously described [36], with a difference in cffDNA yield that corresponded to the difference in plasma equivalent per PCR (extraction input vol.×template vol./elution vol.).

Automated dispensing for the PCR setup took place using the QIAgility instrument (Qiagen Inc.). The QIAgility was then compared with the QIAsymphony AS pipetting robot (Qiagen Inc.) by evaluating each system's effect on detection of fetal and total DNA from parallel DNA extraction and pipetting using 104 paired plasma samples (two plasma samples taken from 104 individuals).

DNA extraction from human plasma reference material 07/222 from NIBSC [37] was performed with the QIAsymphony SP; the PCR setup was manual. The manufacturer's instructions were followed for reconstituting freeze-dried plasma for 5 min at room temperature.

### Real-time PCR

Eluted DNA was analyzed with the real-time PCR ABI 7500 detection system (Applied BioSystems, Foster City, USA) with Taqman chemistry. The total reaction volume was 25 µL using the Universal Master Mix with uracil-N-glycosylase (UNG), (Applied BioSystems). The PCR assay targeted the RhD gene, *RHD* (accession number NT_004610.19). Primers and probes targeting *RHD* exons 7 and 10 were set up in a duplex with both probes labeled with FAM, which provided increased sensitivity [36]. The total DNA was detected by targeting the gene for glyceraldehyde 3-phosphate dehydrogenase (*GAPDH*). The sequences for primers and probes were for *RHD* exon 7 [38]: *RHD*ex7s: 5′-cagctccatcatgggctacaa-3′, *RHD*ex7a: 5′-ccggctccgacggtatc-3′, *RHD*ex7p: 5′-FAM-cagcacaatgtagatgatctctccaagcag-TAMRA-3′; for *RHD* exon 10 [12]: *RHD*ex10s: 5′-cctctcactgttgcctgcatt-3′, *RHD*ex10a: 5′-agtgcctgcgcgaacatt-3′, *RHD*ex10p: 5′-FAM-tacgtgagaaacgctcatgacagcaaagtc-TAMRA-3′; and for *GAPDH* [28]: *GAPDH*s: 5′-ccccacacacatgcacttacc-3′, *GAPDH*a: 5′-cctagtcccagggctttgatt-3′, *GAPDH*p: 5′-FAM-aaagagctaggaaggacaggcaacttggc-TAMRA-3′. Amplicon lengths were 93 base pairs (bp) for *RHD* exon 7, 74 bp for *RHD* exon 10, and 94 bp for *GAPDH*. All oligos were synthesized by Eurofins MWG (Eurofins MWG Operon, Edersberg, Germany) with HPLC purification. The final primer concentration was 900 nM, and the final probe concentration was 100 nM.

DNA samples were analyzed for *RHD* in triplicate reactions with a 10-µL template volume and for *GAPDH* in a single reaction with a 5-µL template volume. The plasma equivalent per PCR was 167 µL for *RHD* detection. Each PCR reaction plate was run with control samples with DNA from *RHD* positive and *RHD* negative individuals. One *RHD* positive control, designated C1, was a single reaction of 500 picograms (pg) DNA per PCR; another *RHD* positive control, designated C2, consisted of triplicate reactions of 50 pg DNA per PCR (C2a–c). Multiple controls without template DNA were also included using sterile H_2_O. The thermal profile consisted of 2 min at 50°C and 10 min at 95°C, followed by 45 cycles of 95°C for 15 sec and 60°C for 1 min.

Real-time PCR results were evaluated with 7500 System SDS software Version 1.4 (Applied BioSystems) using a fixed threshold of 0.2 ΔRn. A PCR reaction was considered positive if the cycle threshold (Ct) value was <41. The criterion for ‘screen positive’ was three positive reactions out of three (3/3); ‘screen inconclusive’ was defined as 2/3 reactions (less than 1% of positive results [18]) or if suspected maternal variant *RHD*, and ‘screen negative’ was defined as 0–1/3 reactions with a positive *GAPDH* at Ct<35. The limit of detection (LOD) for *RHD* was theoretically estimated as an average of 1 DNA copy per PCR reaction with the criterion of 2/3 reactions and 2 copies per PCR reaction with the criterion of 3/3 reactions for a screen positive result.

### DNA quantification

We wanted to examine two different methods for quantifying cffDNA: 1) absolute quantification using a universal standard curve that was not included on the individual PCR plate versus 2) relative quantification using the ΔCt method with a known quantity of reference DNA present on each PCR plate.

For practical and economic reasons, DNA quantification was not performed routinely during each PCR run. Therefore, we constructed a universal standard curve and tested whether this curve could be used to quantify cffDNA based on Ct values from the screening results. The universal standard curve was created from six different standard curves run on different days [39], and a conversion factor of 6.6 pg per genome equivalent (geq) was used to convert pg into geq, thus enabling translation of the mean *RHD* Ct values into gene copies per mL. Standard curve dilutions ranged from 25 pg to 1000 pg per PCR, covering the expected range of cffDNA. Based on Ct values from the six curves, the standard curve characteristics were as follows: y = −3.62x+41.74, R^2^ = 0.924, PCR efficiency = 0.89. See Dataset S1, for further details on the universal standard curve for *RHD*. A universal standard curve for *GAPDH* was also constructed. Based on mean Ct values from the three curves, the standard curve characteristics were: y = −3.52x+40.84, R^2^ = 1, PCR efficiency = 0.92.

To quantify *RHD*, we evaluated the ΔCt method as the second method for cffDNA quantification. We used the set of controls (C1 and C2a–c) that were run simultaneously with the *RHD* analysis of blood samples on each PCR plate. Both C1 and C2 were of known DNA concentrations that were quantified by spectrophotometry, as previously described [33]. We used the mean Ct value for C2a–c to estimate the amount of DNA in C1 using [C1] = 2^ΔCt^*[C2]. Overall, 160 PCR plates were tested.

For further quantification of cffDNA from maternal plasma, we included only results that were *RHD* screen-positive. We excluded blood samples if a positive *RHD* signal was caused by maternal weak D or pseudogenes; results from twin pregnancies and pregnancies with chromosomal abnormalities or fetal malformations were also excluded. A total of 1317 blood samples were included for further quantitative analysis.

### Blood sample transportation time

We investigated whether blood sample transportation time would affect the quantities of fetal and total DNA assessed by real-time PCR-based *RHD* and *GAPDH* detection at GA 25 weeks. Transportation time was defined as the time from blood sampling to the time of DNA extraction measured in full days (no freezing steps were included before DNA extraction). During Jan and Feb 2010, the transportation time was recorded for 110 samples, 52 of which were *RHD* positive. The storage and shipment conditions prior to arriving at the laboratory were unknown.

### Effect of ambient outdoor temperatures on detecting cffDNA

From The Data and Climate Division of the Danish Meteorological Institute (DMI), we retrieved information about the weather in 2010. Average, minimum, and maximum temperatures from each 24-hour day in the Capital Region of Denmark were obtained. Information on transportation time for individual samples was unavailable, but the average transportation time was 4–5 days. Therefore, we calculated a 5-day average of the 24-hour average temperatures for each sample for the 5 days prior to the day of sample arrival. These 5-day averages were correlated with the Ct values of *RHD* and *GAPDH*.

Approximately one third of the blood samples were packed in a Thermo Scientific Nunc Transport Container (Thermo Fisher Scientific Inc., Waltham, MA) and shipped by mail in a padded envelope. Approximately two thirds of the blood samples were transported by car in Smartstore™ Classic 10 (34×25×16 cm) plastic storage boxes with lid (Hammerplast Medical AB, Lidköping, Sweden), where the samples were placed in a foam rack.

### Statistical analysis

For the analysis of blood sample transportation time, we used one-way ANOVA to evaluate the differences between the Ct values of *RHD* and *GAPDH* on different days. The effect of ambient outdoor temperatures was also evaluated using one-way ANOVA; we used an unpaired *t*-test to evaluate differences between temperatures and between Ct values (using Welch's correction for the *t*-test of Ct values). PCR dispensing systems were evaluated using the paired *t*-test. For DNA quantification, we used the unpaired *t*-test to compare precision of each quantification method.

P values<0.05 were considered significant. All statistical calculations were performed using either SPSS (Version 18.0; SPSS Inc., Chicago, IL) or GraphPad Prism 5.2 (GraphPad Software, Inc., San Diego, CA).

## Results

### Blood sample transportation time

**Figure 1:**
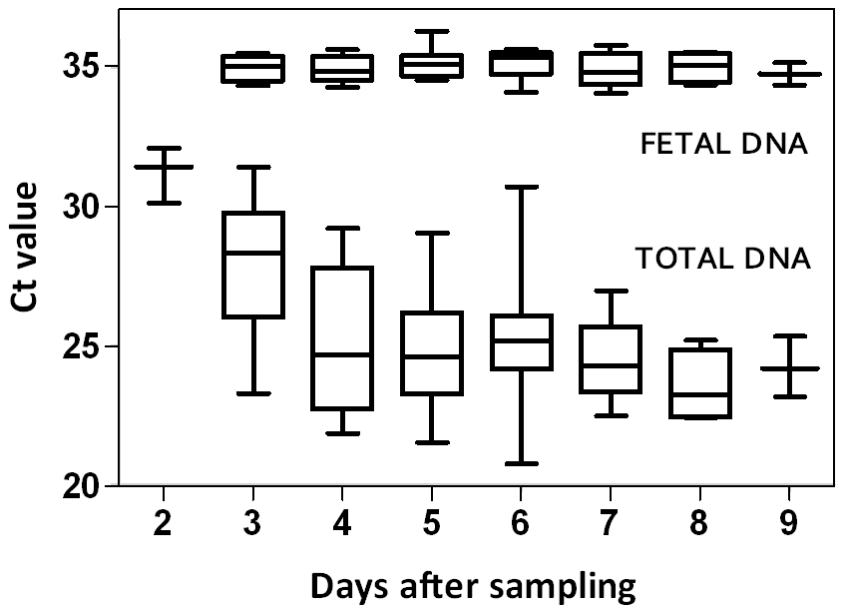
Effects of blood sample transportation time on DNA levels in maternal plasma. The detection of DNA extracted from maternal plasma taken from blood samples (collected in 6-mL EDTA-tubes) subjected to 2–9 days of transport. DNA quantities are shown as Ct values. The total DNA concentration was assessed by detecting *GAPDH* (n = 110). Fetal DNA concentration was assessed by detecting *RHD* (n = 52). The boxes represent 25–75%, the line represents the median Ct values, and the whiskers are minimum and maximum values. The number of samples used to detect fetal and total DNA concentrations (n = fetal/total) was as follows: Day 2 (n = 0/3), Day 3 (n = 4/10), Day 4 (n = 8/17), Day 5 (n = 10/20), Day 6 (n = 12/29), Day 7 (n = 12/20), Day 8 (n = 4/8), and Day 9 (n = 2/3).

Fetal and total DNA levels were measured in plasma from blood samples subjected to 2–9 days of transportation. CffDNA was readily detected regardless of the transportation time and despite an elevation in total DNA (Figure 1). A mean value of approximately 65 copies of cffDNA per mL of maternal plasma was detected throughout the test period of 3–9 days of transport. There was no significant difference between the fetal levels across the period (p = 0.898). The amount of total DNA increased considerably during the first 2–4 days but remained stable from 4–9 days (Figure 1).

### Effect of ambient outdoor temperatures on detecting cffDNA

**Figure 2:**
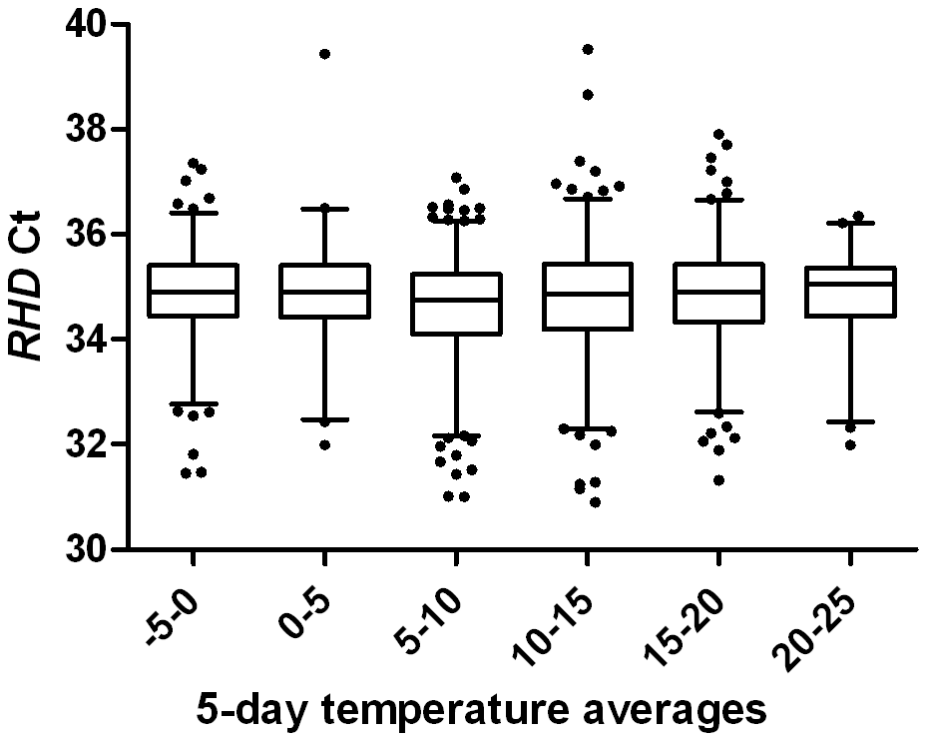
Effect of ambient outdoor temperatures on fetal DNA in maternal plasma. Fetal DNA is shown as *RHD* Ct values against groups of 5-day averages of average daily temperatures, in degrees Celsius. A total of 1539 samples with *RHD* positive result were divided into groups based on 5-day averages of average daily temperatures, −5–0°C (n = 264), 0–5°C (n = 83), 5–10°C (n = 440), 10–15°C (n = 370), 15–20°C (n = 295), and 20–25°C (n = 87). See the Materials and Methods section for a detailed description of temperature calculations. Line inside box, median; limits of box, 75^th^ and 25^th^ percentile; whiskers, 2.5^th^ and 97.5^th^ percentiles.

In this study of ambient outdoor temperatures from 2010, the minimum temperature was −10°C, and the maximum temperature was 28°C. The effect of ambient outdoor temperatures during the transportation of maternal blood samples was tested for 1539 samples that were positive for fetal *RHD*. Divided into different temperature groups, there were no significant differences between the levels of cffDNA, with the exception of the interval of 5 to 10°C compared with −5 to 0°C or 15 to 20°C (p<0.05); see Figure 2.

**Figure 3:**
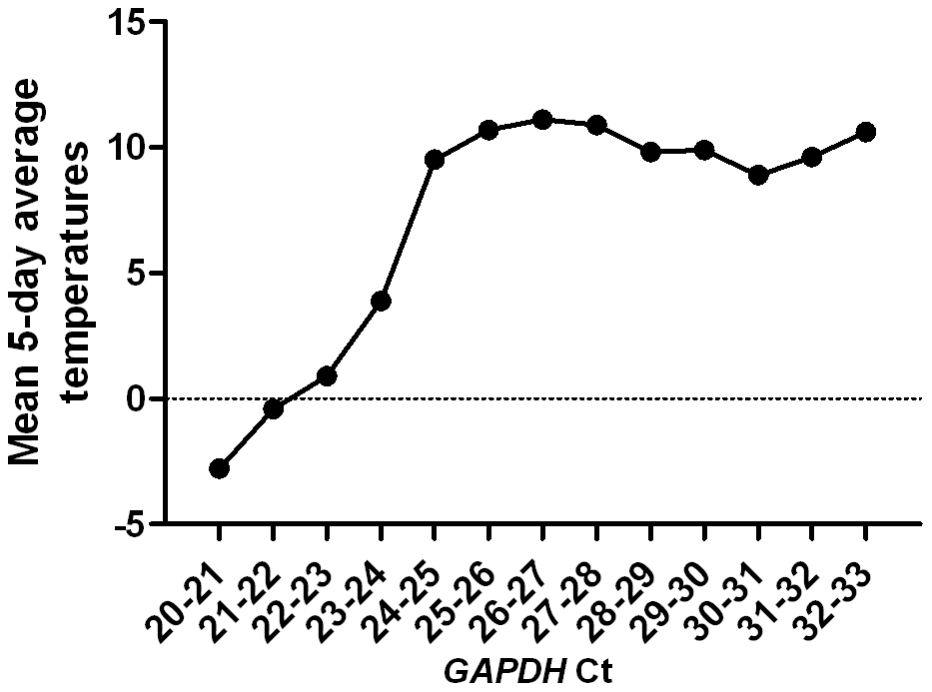
Effect of ambient outdoor temperatures on the total DNA level in maternal plasma. The effect of ambient outdoor temperatures is shown as the mean average degrees Celsius calculated for samples grouped in *GAPDH* Ct value intervals. See the Materials and Methods section for a detailed description of temperature calculations. Neither the mean average temperatures in intervals 20–24 nor those in intervals 24–33 were significantly different from each other. However, the mean average temperatures in intervals 21–24 were significantly different from the mean average temperatures in intervals 24–30.

The samples were then grouped by *GAPDH* Ct values into set intervals, starting at the first interval of Ct 20–21 and ranging to Ct 32–33. For each group, a mean 5-day average of daily average temperatures was then calculated and displayed against each group; see Figure 3. ANOVA analysis revealed that the intervals could be divided into two distinct groups. The first group consisted of samples with *GAPDH* Ct 21–24 (*n* = 108), and the second group consisted of samples with *GAPDH* Ct 24–30 (*n* = 1137). The mean 5-day average of average daily temperatures (2.4°C) in the first group was significantly different from the mean 5-day average of average daily temperatures (10.3°C) in the second group (p<0.0001). The mean *RHD* Ct values (± SEM) were 34.9±0.08 in Group 1 and 34.8±0.03 in Group 2, with no significant difference (p = 0.1522).

### PCR reaction setup

A comparison of the two automated PCR setup systems showed no significant difference between fetal DNA detection as a consequence of changing the dispensing system (p = 0.0595, *n* = 66 pairs). However, total DNA detection was significantly different, p<0.0002 (*n* = 104), and the mean difference corresponding to the amount of total DNA detected using the QIAgility for dispensing was 1.2 times the amount of total DNA detected using the QIAsymphony AS (see Figures S1 and S2).

### Quantification of cffDNA

We compared two quantification methods by quantification of the DNA amount in C1 with a known quantity of 500 pg. We found that the ΔCt method estimated a mean amount of 608 pg (SD 185) in C1, and the universal standard curve estimated a mean amount of 503 pg (SD 145). The mean difference between the estimated results and C1 was −99 pg (p<0.001) for the ΔCt method and 5.7 pg (p = 0.622) for the standard curve. In addition, the universal standard curve estimated the known amount of 50 pg in C2 to a mean of 54 pg (SD 16.6).

**Figure 4:**
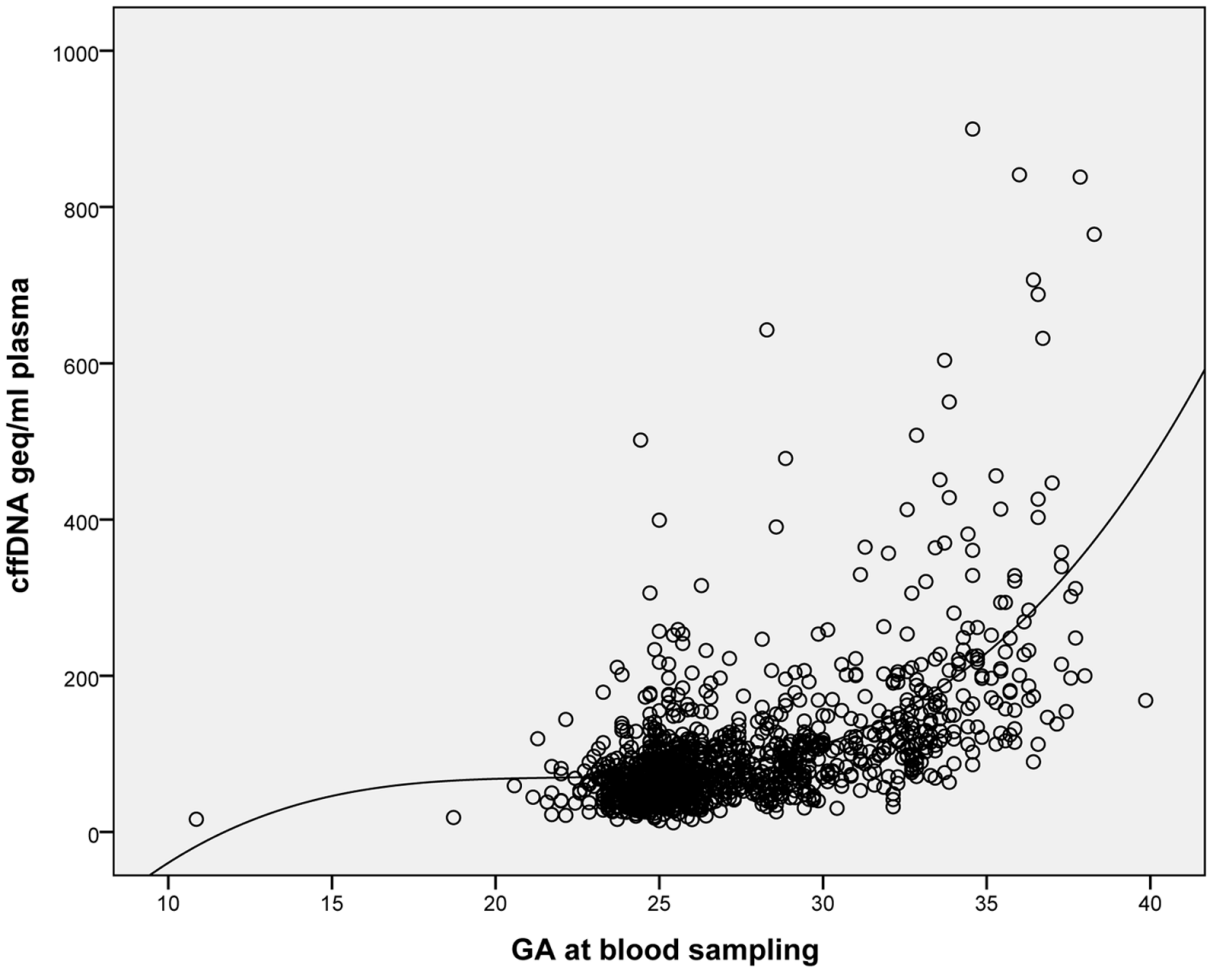
Levels of cffDNA during pregnancy. The cffDNA levels were estimated by the universal standard curve; the cffDNA levels increased with increasing GA at blood sampling. The data shows a marked increase after GA 30 weeks.

Further quantification of cffDNA was then performed with the universal standard curve in 1317 samples. Mean fetal geq/mL plasma was 100 (95% CI: 93–107), with a range of 12–3420. At GA 23–28 weeks, representing GA 25 weeks, the mean fetal geq/mL was 78. The amount of cffDNA increased with increasing GA, as shown in Figure 4. For each sample, we also examined the range of the triplicate Ct values by subtracting the lowest Ct value from the highest Ct value. The mean Ct range was 0.453 Ct (SD 0.387). We found a statistically significant positive correlation between increasing mean Ct value and range (p<0.001).

The mean Ct value for *GAPDH* was 27.52 (95% CI: 27.39–27.64) with a range of 20.74–33.15, equivalent to a mean total DNA value of 6 ng per PCR and a range of 0.2–518 ng per PCR. In all experiments, all positive controls were positive, all negative controls were negative, and all NTCs were negative.

### Test of plasma reference material

Eluted DNA extracted from reconstituted plasma material (07/222 from NIBSC) was diluted 1∶2 and 1∶4. These dilutions were tested by monoplex exon 7, monoplex exon 10, and duplex exon 7/10 in triplicate PCR for each assay. DNA diluted to 1∶2 was amplified by the monoplex exon 7 in two replicates with a mean Ct value of 39.2, by the monoplex exon 10 in all replicates with a Ct value of 40.5, and by the duplex in all replicates with a mean Ct value of 38.5. DNA diluted to 1∶4 was amplified by the monoplex exon 7 and the monoplex exon 10 in only one replicate each, with Ct values of 39.2 and 41.5, respectively; the duplex amplified all replicates with a mean Ct value of 38.7.

### Clinical aspects of antenatal *RHD* screening

**Figure 5:**
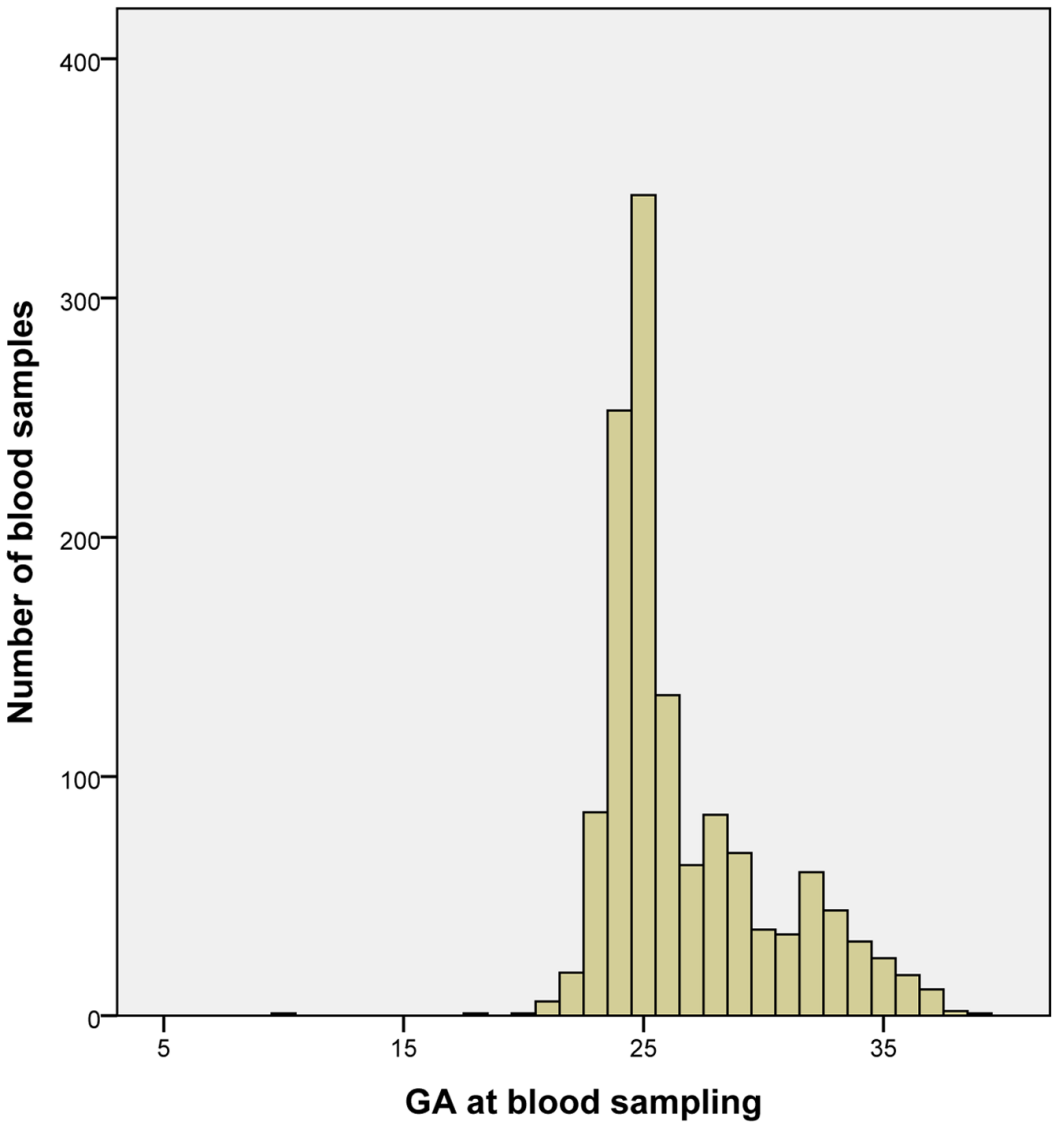
Distribution of blood samples according to GA at sampling. The proper GA at sampling is scheduled at 25 weeks. GA ranged from 10 to 39 weeks.

In the Danish Rh-prophylaxis program, the blood samples for antenatal *RHD* screening analysis are scheduled at GA 25 weeks. The blood samples were taken at a median of GA 25 weeks (range 10–39; Figure 5), and 73% of the samples were taken at GA 23–28 weeks. Only three samples were taken earlier than GA 20 weeks.

We attempted to analyze compliance to the recommendations based on the results from the antenatal *RHD* screening; however, reliable data could not be obtained because of insufficient clinical registration routines.

## Discussion

We conducted a step-by-step evaluation of the laboratory setup for antenatal *RHD* screening in the Capital Region of Denmark and showed that a reliable routine setup is possible for non-invasive detection of fetal *RHD* in D negative women. We found that the transportation of blood samples for up to 9 days affected neither the levels of cffDNA nor the detection of cffDNA, despite a substantial elevation in total DNA. The highest level of total DNA was estimated at approximately 500 ng per PCR. During transportation, only cold 5-day average ambient outdoor temperatures near and below 5°C correlated with high levels of total DNA above 75 ng per PCR. Importantly, we expect a difference between the ambient temperatures and the temperatures that the blood samples were actually exposed to, due to package insulation and/or postal service storage conditions. It is very likely that the blood samples were protected to some extent from the ambient temperatures either partly or fully during the transportation, thus limiting the effect of the ambient temperatures. However, the detection of fetal *RHD* was robust.

In the study of transportation time, approximately 65 copies per mL were detected regardless of a transportation time of 3–9 days. The study did not include samples from 0–2 days of transportation time, and it is possible that the level of cffDNA was higher immediately after sampling and subsequently dropped to the level observed from Day 3. However, several studies have shown that cffDNA is also stable within the first 24 hours [31], [40], [41]. It cannot be excluded that the presence of total DNA decreased the cffDNA to an equal level from Days 3–9. Stable levels of total DNA after 2 days might indicate that all DNA had been released from all maternal cells present in the samples. In an extensive study of the effect of transportation on plasma DNA levels, Müller and colleagues found that cffDNA was stable in blood samples for up to 5 days of transportation [30]. Taken together, these findings are important for laboratories that anticipate prolonged transportation of blood samples in relation to antenatal *RHD* screening. As a consequence of these findings, we have decided to accept samples that are up to 7 days old for analysis. However, we still require that the anticipated transportation time from the general practitioner must not exceed a maximum of 4 days so that the transportation time does not exceed 7 days when samples are in transit over weekends or public holidays.

If next-generation sequencing (NGS) analysis is to be undertaken, the proportion of cffDNA is important, and the transportation results emphasize the importance of using specialized blood-collection tubes to stabilize the level of total DNA [31], [42] and/or reducing the time between sampling and the separation of plasma and cells. We have introduced NGS-based antenatal Kell typing using EDTA tubes [43].

For cffDNA quantification, we found that the universal standard curve was more accurate than the ΔCt method for estimating the amount of DNA in the control sample. The comparison was made using only one DNA concentration (50 pg/µL), thus the difference in the quantitative precision of the two methods at lower DNA concentrations is unknown. This limitation may also have influenced the precision of the ΔCt method. If we incorporated the estimated PCR efficiency into the ΔCt method (as [C1] = 1.89^ΔCt^*[C2]), then the mean difference between the estimated results and C1 was −46.8 pg (as opposed to −99 pg), but was still significantly different from C1 (p<0.001). The ΔCt method used known DNA concentrations run on every PCR plate, and the purpose of including a control with known DNA on every plate is to take into account possible daily variation between the PCR runs. The universal standard curve was constructed from data from six standard curves to obtain a good overall coverage of expected variation. DNA ranged from 25 pg to 1000 pg per PCR reaction, thus also covering small amounts of DNA. Consequently, the R^2^ value was lower than described previously [36]. Although the universal curve was not included simultaneously with the PCR analysis of samples but used merely as a mathematical equation, it was an acceptable solution for quantification. This was also demonstrated by the estimation of the amount of DNA in C2, at the level of 50 pg per PCR. However, quantification of samples with low levels of DNA is best viewed as semi-quantitative.

We found an increase in cffDNA with increasing GA, which is consistent with prior studies [13], [25], [29], [44]. The quantification of cffDNA may play an important role in the detection of severe pregnancy complications, and we have shown that high cffDNA levels are found in women who later develop preeclampsia or deliver preterm [39], [45].

Comparing the estimated number of copies per mL in samples and the LOD indicates good assay robustness for antenatal screening for fetal *RHD* (e.g., an approximately 10-fold distance from a mean of 62 copies per mL at GA 21–25 weeks to a LOD of 6 copies per mL). To ensure the high sensitivity of the antenatal screening, we have set a limit of GA 20 weeks as the earliest acceptable sample collection time.

The real-time PCR setup was based on our original design from 2005 and duplexed as described in 2010 [36], [38]. The duplex results in a higher sensitivity that is equivalent to increasing the DNA targets in the DNA template [36]. The higher sensitivity of the duplex was also demonstrated with the results from the NIBSC plasma reference, despite high Ct values that we believe resulted from poor plasma reconstitution. We previously reported a sensitivity of 100% for the antenatal *RHD* screening [18]. This antenatal *RHD* screening has now been used as a clinical test for more than three years, and screening has included more than 10,000 individuals with no more than 3 false-negative results according to postnatal serology, and postnatal serology is not in itself a 100% reliable [17].

The detection of cffDNA did not significantly differ between the two automated PCR setup systems. Overall, we consider the automated DNA extraction and PCR setup from QIAsymphony SP/AS essential contributors to the accuracy and robustness of our assay.

Blood samples were collected by the general practitioner. The GA was between 10–39 weeks as previously observed [46], which may result from the fact that this analysis and accompanying procedure was recently implemented. However, 73% of the samples were taken at GA 23–28 weeks, which is an improvement from the previously observed 63% figure from the first half of 2010 [46]. Nevertheless, our aim is for more than 95% of the samples to be collected at GA 23–28 weeks. Timely blood sampling is an important goal for avoiding false-negative results and achieving the desired anti-D coverage of the antenatal prophylaxis. Blood samples should be sent for analysis even when collected later than recommended. We were not able to obtain reliable information about compliance for the administration of the antenatal prophylaxis recommended by the results of antenatal screening. A previous minor-scale assessment of compliance from early 2010 showed a compliance of 86% [46], and this figure is anticipated to have improved. We roughly estimate that 90–95% of the expected samples arrive for antenatal *RHD* analysis [46]. Thus, a future task is to assess and further improve compliance.

This study aimed mainly to address pre-analytical issues related to assay sensitivity and robustness. The Rh blood group system is complex [47]. Therefore, it is important to carefully consider the fetal *RHD* target and how to handle maternal and fetal variants that may complicate the accurate prediction of the fetal RhD status in D negative women [47]. We have chosen to target *RHD* exon 7 and 10 because of the possibility of increasing sensitivity, although including exon 10 does produce a limited number of false-positive results. However, when fetal D prediction is used to guide targeted antenatal prophylaxis, false-positive cases are of minor clinical concern, whereas false-negative results must be avoided by all means.

Our setup also allows for further serological and genetic analysis of pregnant women in cases of suspected maternal *RHD* positivity. For such cases, the appropriate action is to recommend antenatal prophylaxis because the fetal *RHD* is masked by the maternal DNA. Future inexpensive sequencing techniques might be used to assess the fetal RhD type in cases with maternal *RHD* variants.

## Conclusion

We evaluated the setup for the routine, non-invasive testing and quantification of cell-free fetal DNA in the Capital Region of Denmark. We showed that transportation time and ambient outdoor temperatures did not affect cffDNA detection. Quantification was possible using a universal standard curve. For antenatal screening for fetal *RHD* at GA 25 weeks, the setup is robust.

## Supporting Information

Dataset S1 **The universal standard curve for** ***RHD*** **.**
(DOCX)

Figure S1 **Comparison of two PCR dispensing systems evaluated by detection of fetal DNA.** Levels of cffDNA shown as *RHD* Ct for two different, automated PCR dispensing systems for PCR setup, the QIAgility and the QIAsymphony Assay Setup (AS) instruments (n = 66 sample pairs). There was no significant difference between the mean levels of cffDNA (p = 0.0595). Line inside box, median; limits of box, 75^th^ and 25^th^ percentile; whiskers, 2.5^th^ and 97.5^th^ percentiles.
(TIF)

Figure S2 **Comparison of two PCR dispensing systems evaluated by detection of total DNA.** Levels of total DNA shown as *GAPDH* Ct for two different, automated PCR dispensing systems for PCR setup, the QIAgility and the QIAsymphony Assay Setup (AS) instruments (n = 104 sample pairs). The mean levels of total DNA were significantly different (p<0.0002). Line inside box, median; limits of box, 75^th^ and 25^th^ percentile; whiskers, 2.5^th^ and 97.5^th^ percentiles.
(TIF)
