# Supplementary material for: Pre-Analytical Conditions in Non-Invasive Prenatal Testing of Cell-Free Fetal RHD
Source: PLoS One. 2013 Oct 18;8(10):e76990. doi: 10.1371/journal.pone.0076990 (PMC3800077; doi:10.1371/journal.pone.0076990)
Supplement: Dataset S1 — The universal standard curve for RHD . (DOCX) [file pone.0076990.s001.docx]

**Dataset S1**

**The universal standard curve for *RHD***

The universal standard curve was based on six different standard curves. The characteristics of the universal standard curve were as follows: y = -3.62x + 41.74, R^2^ = 0.924, PCR efficiency = 0.89.

Below is presented the Ct-values and DNA concentrations of each point calculated by the universal standard curve. As defined by a maximum CV of 25%, the limit of quantification (LOQ) was between 250 pg and 50 pg per PCR, equivalent to 38 and 7.6 copies per PCR. LOQ was further estimated to 150 pg per PCR, equivalent to 22.7 copies per PCR.

| Ct-values from the universal standard curve and calculated DNA concentration parameters | | | | | | | | | | | | |
| --- | --- | --- | --- | --- | --- | --- | --- | --- | --- | --- | --- | --- |
|  |  |  |  |  |  |  |  |  |  |  |  |  |
| **pg/PCR** | **1000** | **500** | **250** | **50** | **25** |  | **pg/PCR** | **1000** | **500** | **250** | **50** | **25** |
|  |  |  |  |  |  |  |  |  |  |  |  |  |
| **Ct-value** | 31.2 | 32.1 | 33.3 | 36.8 | 35.2 |  | **pg/PCR*** | 816.5 | 459.5 | 220.5 | 22.6 | 62.2 |
|  | 30.9 | 32.0 | 32.8 | 35.6 | 35.7 |  |  | 960.4 | 476.2 | 296.6 | 50.7 | 47.2 |
|  | 31.0 | 32.1 | 32.7 | 35.0 | 39.1 |  |  | 944.1 | 469.6 | 313.1 | 72.3 | 5.3 |
|  | 30.4 | 32.1 | 33.3 | 35.6 | 37.2 |  |  | 1324.9 | 455.2 | 218.5 | 49.5 | 17.7 |
|  | 30.7 | 31.9 | 32.3 | 35.3 | 37.0 |  |  | 1127.2 | 524.1 | 405.9 | 60.8 | 20.9 |
|  | 31.0 | 31.5 | 32.7 | 34.7 | 35.9 |  |  | 909.8 | 685.3 | 318.9 | 88.8 | 40.0 |
|  | 30.8 | 32.1 | 33.2 | 35.8 | 35.6 |  |  | 1048.9 | 469.0 | 229.5 | 44.5 | 49.8 |
|  | 30.7 | 32.1 | 33.6 | 35.5 | 36.1 |  |  | 1130.0 | 458.3 | 177.7 | 52.7 | 36.4 |
|  | 30.8 | 32.3 | 33.4 | 36.2 | 38.1 |  |  | 1042.7 | 409.3 | 203.1 | 34.4 | 10.2 |
|  | 30.7 | 32.0 | 33.1 | 36.1 | 35.9 |  |  | 1127.2 | 489.6 | 245.6 | 35.7 | 41.0 |
|  | 31.0 | 32.0 | 33.0 | 35.5 | 37.5 |  |  | 904.0 | 497.9 | 251.8 | 54.4 | 14.4 |
|  | 30.8 | 31.8 | 33.3 | 34.8 | 36.5 |  |  | 1078.6 | 565.1 | 209.0 | 81.3 | 28.6 |
|  |  | 32.1 | 33.2 | 35.2 | 39.0 |  |  |  | 473.2 | 222.0 | 65.8 | 5.6 |
|  |  | 32.1 | 33.1 | 35.0 | 36.5 |  |  |  | 469.1 | 251.0 | 71.2 | 28.7 |
|  |  | 31.8 | 33.3 | 36.5 | 36.1 |  |  |  | 564.6 | 215.8 | 28.1 | 35.5 |
|  |  | 32.1 | 33.0 | 35.6 | 37.1 |  |  |  | 462.4 | 252.5 | 50.0 | 18.6 |
|  |  | 32.1 | 33.2 | 35.1 | 38.0 |  |  |  | 446.6 | 223.7 | 68.6 | 10.8 |
|  |  | 31.5 | 33.1 | 35.9 | 38.0 |  |  |  | 690.7 | 246.2 | 41.5 | 11.4 |
|  |  |  |  | 34.7 | 35.5 |  |  |  |  |  | 89.8 | 54.6 |
|  |  |  |  | 35.5 | 36.4 |  |  |  |  |  | 54.2 | 29.0 |
|  |  |  |  | 35.4 | 35.6 |  |  |  |  |  | 56.7 | 50.9 |
|  |  |  |  | 35.8 | 37.0 |  |  |  |  |  | 44.3 | 21.7 |
|  |  |  |  | 35.5 | 36.1 |  |  |  |  |  | 52.0 | 35.3 |
|  |  |  |  | 34.9 | 37.0 |  |  |  |  |  | 75.9 | 20.8 |
|  |  |  |  | 35.2 | 36.0 |  |  |  |  |  | 64.9 | 37.8 |
|  |  |  |  | 35.7 | 35.8 |  |  |  |  |  | 46.9 | 44.7 |
|  |  |  |  | 35.2 | 39.0 |  |  |  |  |  | 63.5 | 5.6 |
|  |  |  |  | 35.0 | 37.6 |  |  |  |  |  | 72.1 | 13.5 |
|  |  |  |  | 35.0 | 37.3 |  |  |  |  |  | 71.5 | 16.4 |
|  |  |  |  | 34.8 | 36.9 |  |  |  |  |  | 82.1 | 21.3 |
|  |  |  |  |  |  |  | **Median** | 1045.8 | 471.4 | 237.6 | 55.6 | 25.2 |
|  |  |  |  |  |  |  | **Mean** | 1034.5 | 503.7 | 250.1 | 58.2 | 27.9 |
|  |  |  |  |  |  |  | **SD** | 137.1 | 77.3 | 53.8 | 17.3 | 16.0 |
| *pg/PCR estimated via the universal standard curve | | | | | | | **CV%** | 13.2 | 15.3 | 21.5 | 29.7 | 57.5 |
