# Supplementary figures and images for: Pre-Analytical Conditions in Non-Invasive Prenatal Testing of Cell-Free Fetal RHD
Source: PLoS One. 2013 Oct 18;8(10):e76990. doi: 10.1371/journal.pone.0076990 (PMC3800077; doi:10.1371/journal.pone.0076990)

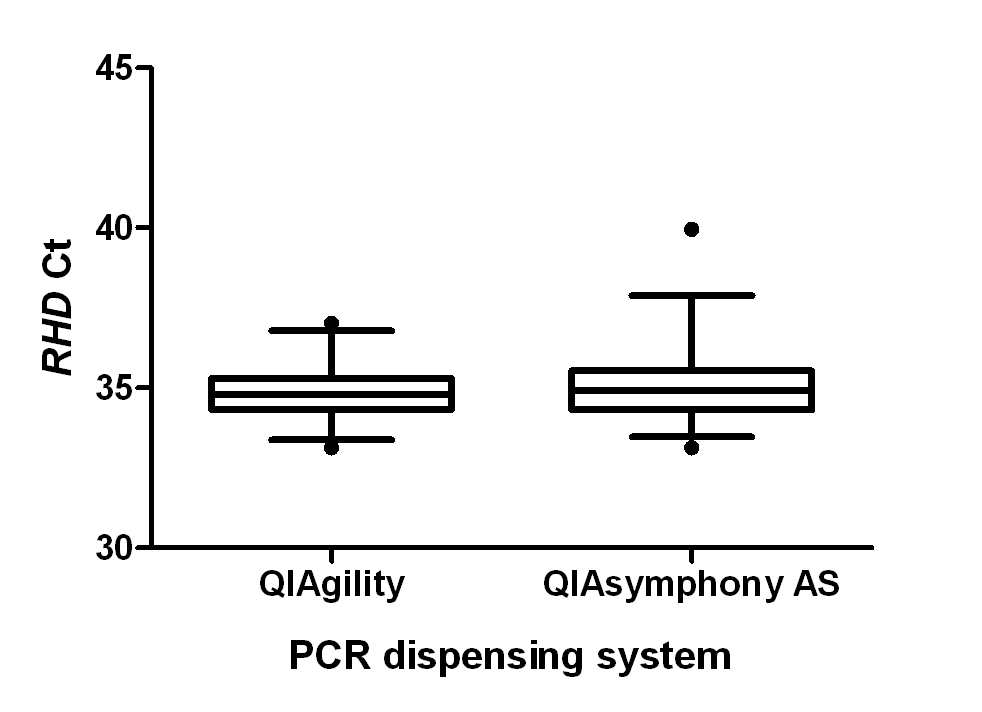

Supplement: Figure S1 — Comparison of two PCR dispensing systems evaluated by detection of fetal DNA. Levels of cffDNA shown as RHD Ct for two different, automated PCR dispensing systems for PCR setup, the QIAgility and the QIAsymphony Assay Setup (AS) instruments (n = 66 sample pairs). There was no significant difference between the mean levels of cffDNA (p = 0.0595). Line inside box, median; limits of box, 75th and 25th percentile; whiskers, 2.5th and 97.5th percentiles. (TIF) [file pone.0076990.s002.tif]

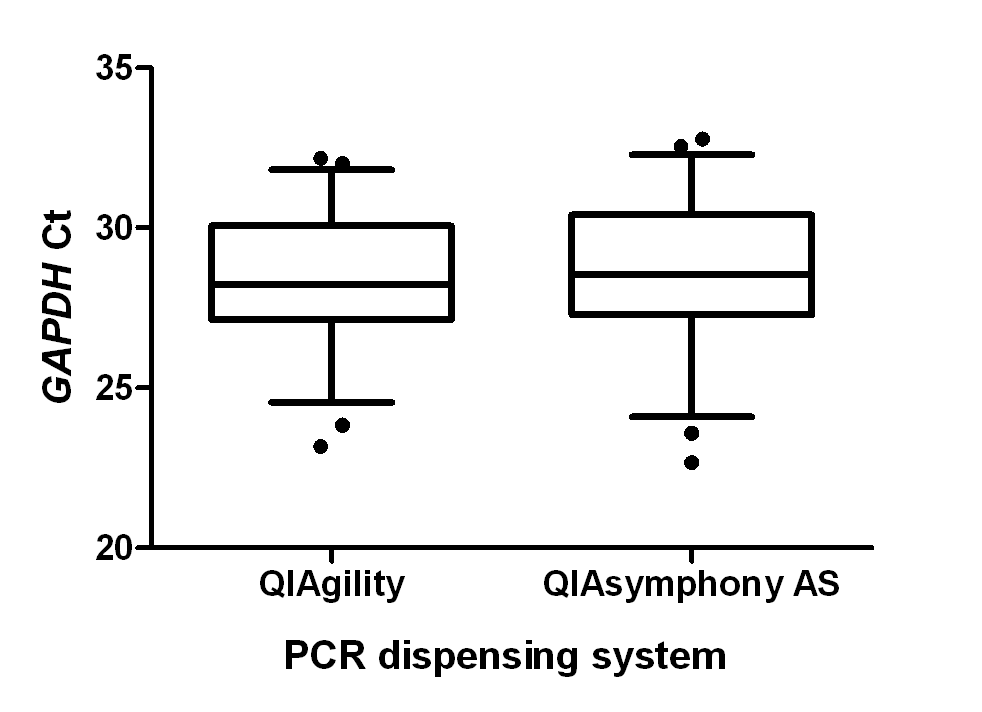

Supplement: Figure S2 — Comparison of two PCR dispensing systems evaluated by detection of total DNA. Levels of total DNA shown as GAPDH Ct for two different, automated PCR dispensing systems for PCR setup, the QIAgility and the QIAsymphony Assay Setup (AS) instruments (n = 104 sample pairs). The mean levels of total DNA were significantly different (p<0.0002). Line inside box, median; limits of box, 75th and 25th percentile; whiskers, 2.5th and 97.5th percentiles. (TIF) [file pone.0076990.s003.tif]
